# Supplementary material for: Risk Group Stratification for Recurrence-Free Survival and Early Tumor Recurrence after Radiofrequency Ablation for Hepatocellular Carcinoma
Source: Cancers (Basel). 2023 Jan 22;15(3):687. doi: 10.3390/cancers15030687 (PMC9913840; doi:10.3390/cancers15030687)
Supplement: Supplementary file 1 [file cancers-15-00687-s001.zip › cancers-2144689-supplementary.pdf]

**Supplementary Table S1. Formulas for the Albumin-Bilirubin Score, Aspartate Aminotransferase/platelet Ratio Index, "Model for Tumor Recurrence after Living Donor Liver Transplantation" (MoRAL) Score, and High Risk of Microvascular Invasion**

| | |
|---|---|
| ALBI score | [log 10 bilirubin (µmol/L) × 0.66] + [albumin (g/L) × -0.085] |
| APRI | [AST (IU/L) / upper limit normal of AST/PLT (× $10^9$/L)] × 100 |
| MoRAL score | 11×√[PIVKA-II] +2×√ [AFP] |

ALBI = albumin-bilirubin, APRI = aspartate aminotransferase/platelet ratio index, MoRAL = Model for tumor recurrence after living donor liver transplantation.

**Supplementary Table S2. Scoring System to Predict High Risk of Microvascular Invasion**

| Serum alpha-fetoprotein level (ng/mL) | | Points |
|---|---|---|
| | <15 | 0 |
| | ≥15 | 1 |
| Serum protein induced by vitamin K absence-II level (mAU/mL) | | Points |
| | <48 | 0 |
| | ≥48 | 1 |
| Peritumoral parenchymal enhancement on arterial phase | | Points |
| | (-) | 0 |
| | (+) | 1.5 |
| Peritumoral hypointensity on hepatobiliary phase | | Points |
| | (-) | 0 |
| | (+) | 2.5 |
| MVI risk group | | Total points |
| | MVI-high risk group | ≥ 3.5 |
| | MVI-low risk group | < 3.5 |

A scoring system to classify HCCs with a high risk of microvascular invasion (MVI) using alpha-fetoprotein (AFP), protein induced by vitamin K absence-II (PIVKA-II), peritumoral parenchymal enhancement, and peritumoral hypointensity on the hepatobiliary phase (HBP) on liver MRI was calculated. In this model, one point is given to AFP if ≥15 ng/mL, one point to PIVKA-II if ≥48 mAU/mL, 1.5 points if peritumoral arterial enhancement is present, and 2.5 points if peritumoral hypointensity on HBP is present. If the total number of points is ≥3.5, it is regarded as MVI-high risk.

AFP = alpha-fetoprotein, PIVKA-II = protein induced by vitamin K absence-II, HBP = hepatobiliary phase, MVI = microvascular invasion

**Nomogram**

For the nomogram, the variable with the biggest impact in the predictive model was assigned 100 points. Then, points were assigned to other variables according to their relative influence compared with the variable with the biggest impact in the predictive model. To use a nomogram, each parameter scale is first marked according to the used parameter. From the marked point on each parameter scale, the corresponding prognostic point is found on the point scale by drawing a perpendicular line from the parameter scale to the point scale. After adding the prognostic points of all parameters, the probability of the outcome, either recurrence or death for RFS and any kind of recur within 2 years after RFA for early tumor recurrence, is estimated by drawing a perpendicular line from the total point scale to the predictive value scale.

**Supplementary Table S3. Results of Inter-Reader Agreement**

| Variables | Kappa | 95% CI |
|---|---|---|
| Non-rim arterial hyperenhancement | 0.865 | 0.759 - 0.971 |
| Washout appearance | 0.724 | 0.614 - 0.834 |
| Enhancing capsule | 0.622 | 0.495 - 0.749 |
| LR-M | 0.785 | 0.663 - 0.907 |
| Peri-tumoral enhancement | 0.782 | 0.673 - 0.891 |
| Non-smooth margin | 0.682 | 0.562 - 0.801 |
| Peritumoral hypointensity | 0.716 | 0.515 - 0.917 |
| Low SI on HBP (reference=iso/high) | 0.805 | 0.618 - 0.991 |

A κ value of 0.01–0.20 was interpreted as slight, 0.21–0.40 as fair, 0.41–0.60 as moderate, 0.61–0.80 as substantial, and 0.81–1.00 as excellent agreement
